# Age- and sex-specific reference values for physical literacy in Colombian children: a GAMLSS analysis of the CAPL-2

**DOI:** 10.3389/fspor.2026.1963012

**Published:** 2026-09-07

**Authors:** Brayan E. Patiño-Palma, José Armando Vidarte-Claros

**Affiliations:** 1 ZIPATEFI Research Group, Faculty of Health and Sports Sciences, Fundación Universitaria del Área Andina, Pereira, Colombia; 2 Doctoral Program in Health Sciences, Universidad Autónoma de Manizales, Manizales, Colombia

**Keywords:** child, Colombia, physical activity, physical fitness, physical literacy, reference values (MeSH)

## Abstract

**Introduction:**

Physical literacy is a multidimensional construct that integrates physical competence, daily behavior, motivation and confidence, and knowledge and understanding; however, the interpretation of assessment scores may vary across sociocultural contexts. This study aimed to develop age- and sex-specific reference values for the Canadian Assessment of Physical Literacy, second edition (CAPL-2), in Colombian schoolchildren aged 8-12 years.

**Methods:**

A cross-sectional multicenter sample of 843 children from the six geographical subregions of Colombia was assessed using the Colombian Spanish adaptation of the CAPL-2. Generalized additive models for location, scale, and shape were fitted separately by sex to estimate smoothed percentiles for the total score and four domains, with age modeled continuously in the location (*μ*) and scale (*σ*) parameters. Empirical percentiles were also calculated for selected physical assessment components, and the percentile positions of the original interpretive categories were examined.

**Results:**

Physical Competence and total scores increased with age and were generally higher in boys, whereas Motivation and Confidence showed greater stability, particularly among boys. Daily Behavior and several physical assessment components also showed higher values in boys, while Knowledge and Understanding showed comparatively small age- and sex-related differences. Importantly, the official interpretive categories occupied markedly different percentile positions across domains.

**Discussion:**

These findings provide contextual reference values that complement the original classification system and allow CAPL-2 performance to be interpreted relative to children of the same age and sex within a geographically diverse Colombian reference sample, without constituting diagnostic thresholds or nationally representative norms.

## 1 Introduction

Physical literacy has emerged as a multidimensional construct relevant to the promotion of lifelong engagement in physical activity. Rather than referring exclusively to physical fitness or motor proficiency, physical literacy integrates physical competence, motivation and confidence, knowledge and understanding, and the behavioral disposition required to engage meaningfully in physical activity throughout life. This multidimensional perspective has contributed to increasing interest in physical literacy within health, education, sport, and physical activity research, particularly during childhood, when movement experiences and opportunities for participation may contribute to the development of active behaviors across subsequent stages of life (1, 2).

Among the available assessment approaches, the Canadian Assessment of Physical Literacy, second edition (CAPL-2), provides a comprehensive framework specifically designed for children aged 8–12 years. CAPL-2 operationalizes physical literacy through four interrelated domains (Physical Competence, Daily Behavior, Motivation and Confidence, and Knowledge and Understanding) and integrates objective physical assessments, direct monitoring of physical activity, and questionnaire-based measures. The instrument was developed following refinement of the original Canadian Assessment of Physical Literacy to improve feasibility while preserving its multidimensional structure (3, 4). The scoring system produces domain-specific scores and a total physical literacy score ranging from 0 to 100 points, together with four interpretive categories: Beginning, Progressing, Achieving, and Excelling (3).

Since its development in Canada, CAPL-2 has been translated, culturally adapted, or cross-validated in several populations. Studies have reported adaptations or measurement-property evaluations in Chinese, Danish, Spanish, Portuguese, Urdu-speaking and other populations, supporting its applicability across different settings while also highlighting the importance of evaluating the instrument within the sociocultural context in which it is used (5–10). A previous scoping review identified substantial heterogeneity in CAPL-2 validation procedures and highlighted the limited evidence available from Latin American populations (11). Differences in opportunities for physical activity, school environments, participation in organized sport, socioeconomic conditions, and sociocultural characteristics may influence physical literacy-related behaviors and consequently the distribution of CAPL-2 scores across populations. Therefore, scores derived from one population may not necessarily occupy the same relative position when interpreted in another context (5, 11).

This issue is particularly relevant when CAPL-2 scores are interpreted using its original classification system. Although the categories Beginning, Progressing, Achieving, and Excelling provide standardized thresholds for interpreting performance, they do not indicate where an individual child is positioned relative to peers of the same age and sex within a specific population (3, 4). Population-specific reference values may therefore provide complementary information by locating an observed score within an empirical distribution while preserving the original CAPL-2 classification system.

Age and sex are particularly relevant when constructing such reference values. Physical competence, fitness, and habitual physical activity vary throughout childhood, and sex-related differences may emerge or become more pronounced as children approach adolescence. CAPL-2 studies conducted in different populations have similarly reported variation in total and domain-specific scores according to age and sex (5, 6). For example, the cross-validation of the Chinese CAPL-2 identified sex differences in total physical literacy scores, with boys showing higher scores than girls (5). Consequently, reference values stratified by age and sex may provide additional information beyond a single set of absolute score thresholds.

Physical literacy may also be relevant to mental and emotional well-being because its multidimensional structure includes motivational, confidence-related, behavioral, and physical components that may influence how children engage with movement and physical activity. Previous research has suggested that children with behavioral and emotional mental health disorders may present lower levels of several physical literacy-related attributes, including motor proficiency, physical activity participation, motivation, and confidence (12). In parallel, evidence from children and adolescents indicates that higher levels of physical activity are associated with better psychological well-being and lower psychological ill-being, although intervention evidence appears more consistent in adolescents than in children (13). These findings support the potential relevance of physical literacy as a framework for understanding factors associated with children's engagement in health-enhancing physical activity and well-being, while recognizing that the direction and causal nature of these relationships remain to be established.

The construction of reference values requires statistical approaches capable of representing distributions that may change according to age and may exhibit differences in dispersion, skewness, or kurtosis. Generalized additive models for location, scale, and shape (GAMLSS) provide a flexible framework for constructing smoothed centile curves because different parameters of the outcome distribution can be modeled as functions of explanatory variables such as age (14, 15). GAMLSS-based methods have been extensively used to construct pediatric reference distributions and allow the use of flexible distribution families when the outcome does not adequately conform to normality (14, 16).

In Colombia, evidence regarding the assessment of physical literacy using CAPL-2 remains limited. Previous work has identified a marked scarcity of CAPL-2 studies from Latin America and the absence of locally derived reference distributions that allow Colombian children's scores to be interpreted according to age and sex (11). Establishing such reference values from a geographically diverse Colombian multicenter sample could therefore provide a contextual framework for interpreting CAPL-2 performance and complement, rather than replace, the original classification system.

Accordingly, this study aimed to develop age- and sex-specific reference values for the CAPL-2 total score and its four domains in Colombian schoolchildren aged 8–12 years using generalized additive models for location, scale, and shape (GAMLSS). As secondary objectives, the study examined the correspondence between the original CAPL-2 interpretive categories and the Colombian percentile distributions and described empirical age- and sex-specific percentiles for selected raw CAPL-2 components.

## 2 Materials and methods

### 2.1 Study design

This observational, cross-sectional, multicenter study was conducted to develop age- and sex-specific reference values for the Colombian Spanish adaptation of the Canadian Assessment of Physical Literacy, second edition (CAPL-2), in schoolchildren aged 8–12 years.

### 2.2 Participants, sampling, and eligibility criteria

The study population comprised Colombian schoolchildren aged 8–12 years enrolled in public educational institutions located across the six geographical subregions of the country: Caribbean, Pacific, Central Eastern, Central-Southern Amazonian, Coffee-growing-Antioquia, and Plains-Orinoquía.

A multistage sampling strategy combining purposive and random selection procedures was used. Cities and educational institutions were selected purposively according to operational feasibility, institutional authorization, and the capacity to implement the CAPL-2 protocol under standardized conditions. Within each participating institution, eligible schoolchildren were subsequently selected at random.

No separate *a priori* sample size calculation was performed specifically for the construction of the percentile reference values. All eligible participants with available CAPL-2 data were considered for the present analysis, resulting in a reference sample of 843 schoolchildren.

To facilitate territorial comparability within the multicenter sample, recruitment was approximately balanced across the six geographical subregions, with 140 participants from the Caribbean, Pacific, and Central Eastern subregions and 141 participants from the Central-Southern Amazonian, Coffee-growing-Antioquia, and Plains-Orinoquía subregions. This allocation was intentionally balanced and was not proportional to the population size of each subregion. The sample was also balanced by sex, comprising 425 girls (50.4%) and 418 boys (49.6%), with similar representation across the five age groups.

Children were eligible if they were 8–12 years of age at the time of assessment, were enrolled in a participating school, had institutional authorization to participate, and provided assent together with written informed consent from a parent or legal guardian. Children were excluded if a medical or physical condition restricted their safe participation in physical activity or CAPL-2 assessments, if their medical history precluded participation in school-based physical activity, or if a cognitive or communication condition prevented them from understanding the instructions or completing the questionnaires.

Participants with incomplete records were retained in the overall database. Analyses were conducted using the valid observations available for each CAPL-2 domain, total score, or assessment component, without imputation of missing data. Because cities and educational institutions were selected purposively, the resulting percentiles should be interpreted as reference values derived from a geographically diverse multicenter Colombian sample rather than as definitive national population norms.

### 2.3 Instrument

Physical literacy was assessed using a Colombian Spanish adaptation of the Canadian Assessment of Physical Literacy, second edition (CAPL-2), originally developed to assess physical literacy in children aged 8–12 years. CAPL-2 operationalizes physical literacy through four interrelated domains: Physical Competence, Daily Behavior, Motivation and Confidence, and Knowledge and Understanding (3, 4).

Before data collection, the CAPL-2 materials were cross-culturally adapted to Colombian Spanish using a standardized process that included forward translation, reconciliation, back-translation, expert appraisal, and pilot testing in schoolchildren aged 8–12 years. The adaptation focused on semantic, idiomatic, conceptual, and contextual correspondence while preserving the original CAPL-2 domains, assessment components, physical tasks, scoring criteria, and interpretation algorithms. No modifications were made to the measurement structure or scoring system of the instrument.

The Physical Competence domain comprises the Progressive Aerobic Cardiovascular Endurance Run (PACER), the Canadian Agility and Movement Skill Assessment (CAMSA), and the plank isometric hold. These components assess cardiorespiratory endurance, motor competence, and muscular endurance, respectively. The CAMSA was originally developed and evaluated as a field-based assessment of fundamental, complex, and combined movement skills in children aged 8–12 years (15). The Daily Behavior domain includes objectively recorded daily step counts using a pedometer and self-reported participation in physical activity.

The Motivation and Confidence domain assesses predilection, adequacy, intrinsic motivation, and perceived competence related to physical activity. The Knowledge and Understanding domain evaluate knowledge concerning physical activity recommendations, cardiorespiratory fitness, muscular strength and endurance, and movement-related concepts. Together, these domains provide complementary physical, behavioral, affective, and cognitive information related to physical literacy (3, 4).

According to the original CAPL-2 scoring structure, Physical Competence, Daily Behavior, and Motivation and Confidence each contribute a maximum of 30 points, whereas Knowledge and Understanding contributes a maximum of 10 points. Domain scores are summed to produce a total CAPL-2 score ranging from 0 to 100 points. Scores can additionally be interpreted using the four original CAPL-2 categories: Beginning, Progressing, Achieving, and Excelling (3).

CAPL-2 domain scores, total scores, and interpretive categories were calculated according to the original scoring algorithms using the *capl* package for R, which was specifically developed to process raw CAPL assessment data and generate standardized scores and interpretations (17). The use of the Colombian Spanish adaptation in the present study therefore involved linguistic and contextual adaptation of the administration materials while retaining the original CAPL-2 measurement and scoring structure.

### 2.4 Data collection

Data collection was conducted during the regular school day in coordination with the participating educational institutions. Assessments were scheduled in authorized spaces and at agreed times to minimize interference with regular academic activities. Each participant was assigned a unique identification code, which was used to link questionnaire responses, physical assessments, self-reported physical activity, and pedometer records while preserving confidentiality.

The CAPL-2 assessment was organized into four sessions distributed across the school week, following the standardized administration procedures of the original protocol (3). During the first session, participants completed the questionnaire-based components and received a pedometer together with instructions for its use and completion of the corresponding activity log. During the second session, motor competence was assessed using the Canadian Agility and Movement Skill Assessment (CAMSA). The Progressive Aerobic Cardiovascular Endurance Run (PACER) was administered during the third session. During the fourth session, pedometer data were collected and muscular endurance was assessed using the plank isometric hold.

Daily Behavior was assessed using pedometer-recorded daily steps and self-reported participation in physical activity. Physical Competence was evaluated using the PACER, CAMSA, and plank assessments, whereas Motivation and Confidence and Knowledge and Understanding were assessed using the corresponding questionnaire-based components of the CAPL-2 (3, 4).

Field assessments were conducted by trained evaluators who received prior instruction and calibration in the Colombian Spanish adaptation of the CAPL-2. Training included review of the administration protocol, standardization of instructions, completion of data-recording forms, use of the pedometer, questionnaire administration, and implementation of the physical assessments. Similar conditions were maintained across participating institutions with respect to the physical setting, scheduling, initial instructions, participant supervision, and general sequence of assessment.

Data collected across the six geographical subregions were reviewed, coded, and integrated into a consolidated analytical database by CAPL-2 domain and assessment component. Data completeness was subsequently verified for each CAPL-2 domain, total score, and assessment component before statistical analysis.

### 2.5 Statistical analysis

Statistical analysis was conducted in five stages: data cleaning and verification, calculation of CAPL-2 scores, description of the reference sample, estimation of age- and sex-specific reference percentiles using generalized additive models for location, scale, and shape (GAMLSS), and complementary analysis of selected raw CAPL-2 components.

The analytical database was examined for consistency in participant identifiers, age, sex, geographical subregion, and the variables required to calculate CAPL-2 scores. Plausibility and theoretical-range checks were applied before analysis; observations were not excluded solely on the basis of statistical extremeness when they remained within the permissible range of the corresponding assessment. Missing data were not imputed; each analysis was conducted using the valid observations available for the corresponding CAPL-2 domain, total score, or assessment component. CAPL-2 domain and total scores were calculated according to the original scoring structure using the capl package for R (17). Data availability was reported as the number and percentage of valid observations for each domain and the total score.

Age- and sex-specific reference values were estimated using generalized additive models for location, scale, and shape (GAMLSS). Age was included as a continuous explanatory variable, and models were fitted separately for girls and boys. School, city, and geographical subregion were not included as covariates, random effects, or clustering terms in the percentile models. Penalized B-splines were used to model age-related variation in both the location (μ) and scale (*σ*) parameters, whereas the shape parameters for skewness (*ν*) and kurtosis (*τ*), when applicable to the selected distribution, were modeled as constants. The final models used low-complexity smooth functions for both μ and *σ*, with approximately two effective degrees of freedom in the fitted age terms. This specification allowed the central tendency and dispersion of CAPL-2 scores to vary smoothly across age while avoiding unnecessarily complex age functions.

For each CAPL-2 domain and the total score, four candidate distribution families were evaluated: normal (NO), Box-Cox Cole and Green (BCCG), Box-Cox power exponential (BCPE), and Box-Cox t (BCT). Models were fitted using the RS algorithm. Candidate models were assessed according to successful convergence, the generalized Akaike information criterion (GAIC), complementary examination of the Schwarz Bayesian criterion (SBC), and diagnostic adequacy based on normalized quantile residuals and worm plots (14, 15, 18). When the initial iteration limit was insufficient, the maximum number of iterations was increased to verify convergence. Final model selection therefore considered both information criteria and diagnostic performance rather than relying exclusively on the lowest GAIC. The selected models were used to estimate age- and sex-specific percentiles for each CAPL-2 domain and the total score. Percentiles were generated from P5 to P95 in five-percentile increments, with P5, P25, P50, P75, and P95 selected for presentation in the main results. Estimated percentiles were subsequently examined to confirm their logical ordering and consistency with the theoretical score range of each CAPL-2 outcome. Percentile values falling below the theoretical minimum or above the theoretical maximum were restricted to the corresponding score limits. The final percentile tables were also checked to confirm the absence of percentile crossing within each age- and sex-specific distribution. To examine the correspondence between the original CAPL-2 interpretive categories and the reference distributions derived from the Colombian multicenter sample, the minimum score required to enter each official category was identified and its corresponding percentile position was estimated within each age- and sex-specific distribution. The original CAPL-2 classification thresholds were not modified. This analysis was intended to describe the relative position of the official category thresholds within the reference distributions obtained in the present sample.

Finally, empirical percentiles by age and sex were calculated for selected raw CAPL-2 components: average daily steps, PACER laps, plank duration, and CAMSA completion time. These percentiles were calculated directly from the observed data and reported in their original measurement units: steps/day for average daily steps, number of laps for PACER, and seconds for plank and CAMSA. For CAMSA, the shortest completion time across the two attempts was used because shorter times indicate better performance. These empirical percentiles were considered descriptive and complementary and were not treated as substitutes for the GAMLSS-derived reference values for the CAPL-2 domain and total scores.

All analyses were conducted in R using the *capl* package for CAPL-2 score calculation (17) and the GAMLSS framework for percentile modelling (14, 18). To enhance transparency and reproducibility, the complete analytical workflow used to generate the reference values, including data-processing procedures, model fitting, distribution-family comparisons, model selection, and percentile estimation, is publicly available at: https://rpubs.com/brayanpp94/valores-referencia-AF.

### 2.6 Ethical considerations

The study was conducted in accordance with the ethical principles of the Declaration of Helsinki and the Colombian regulations governing health research (19, 20). Ethical approval was granted by the Ethics, Bioethics and Scientific Integrity Committee of the Universidad Autónoma de Manizales (approval code 257-191; February 26, 2025). According to Colombian Resolution 008430 of 1993, the study was classified as minimal-risk research (20).

Written informed consent was obtained from the parents or legal guardians of all participating children, and assent was obtained from the children before participation. Participant confidentiality was maintained throughout data collection, database consolidation, and statistical analysis through the use of unique identification codes.

## 3 Results

### 3.1 Availability of valid CAPL-2 data

The reference sample comprised 843 schoolchildren aged 8–12 years, including 425 girls (50.4%) and 418 boys (49.6%). Participants were similarly distributed across the five age groups. Age- and sex-specific analytical sample sizes were relatively balanced across strata, ranging from 70 to 88 participants per age-sex combination depending on the CAPL-2 outcome; detailed analytical sample sizes are provided in Supplementary Table S5.

The number of valid observations varied across CAPL-2 outcomes because each domain and the total score required complete information from different assessment components. Data completeness was high for all outcomes, exceeding 96% in every case. The highest number of valid observations was available for Knowledge and Understanding, with 839 participants (99.5%), whereas the total CAPL-2 score had the lowest number of complete cases, with 816 participants (96.8%), because its calculation required valid information across all four domains. Percentile models were fitted using the valid observations available for each outcome, without imputation of missing data (Table 1).

**Table 1:** Availability of valid data for the estimation of CAPL-2 reference values.

Domain	Variable	Valid n	Valid %
Physical competence	pc_score	829	98.3
Daily behavior	db_score	819	97.2
Motivation and confidence	mc_score	834	98.9
Knowledge and understanding	ku_score	839	99.5
Total CAPL-2 score	capl_score	816	96.8

Percentages were calculated using the total reference sample of 843 schoolchildren as the denominator. Valid cases correspond to participants with complete information required to calculate each CAPL-2 domain or the total score. Missing values were not imputed.

### 3.2 Selected GAMLSS models

Once the valid analytical dataset had been defined for each CAPL-2 score, GAMLSS models were fitted separately for girls and boys, with age included as a continuous explanatory variable. Four candidate distribution families were evaluated for each domain and for the total CAPL-2 score: normal (NO), Box-Cox Cole and Green (BCCG), Box-Cox power exponential (BCPE), and Box-Cox t (BCT). Final models were selected by considering successful convergence, GAIC, complementary SBC values, and diagnostic adequacy based on normalized quantile residuals and worm plots. All retained models achieved convergence. In cases where the lowest-GAIC model showed inadequate diagnostic behavior, preference was given to a convergent alternative with acceptable residual and graphical diagnostics. The selected models for each domain and sex are presented in Table 2.

**Table 2:** Selected GAMLSS models for the estimation of CAPL-2 reference percentiles by domain and sex.

Domain	Variable	Sex	n	Selected model	GAIC	SBC
Physical competence	pc_score	Girls	417	BCCG	2452.32	2472.49
Physical competence	pc_score	Boys	412	NO	2638.96	2655.04
Daily behavior	db_score	Girls	414	BCPE	2614.74	2638.90
Daily behavior	db_score	Boys	405	BCPE	2640.15	2664.17
Motivation and confidence	mc_score	Girls	419	BCPE	2341.18	2365.41
Motivation and confidence	mc_score	Boys	415	BCCG	2352.31	2372.45
Knowledge and understanding	ku_score	Girls	423	NO	1997.36	2013.55
Knowledge and understanding	ku_score	Boys	416	NO	1963.13	1979.25
Total CAPL-2 score	capl_score	Girls	413	BCPE	3174.44	3198.58
Total CAPL-2 score	capl_score	Boys	403	NO	3068.57	3084.57

NO, normal distribution; BCCG, Box-Cox Cole and Green; BCPE, Box-Cox power exponential; BCT, Box-Cox t; GAIC, generalized Akaike information criterion; SBC, Schwarz Bayesian criterion. Model selection considered convergence, information criteria, and diagnostic adequacy. The complete comparison of candidate models and model diagnostics is provided in the Supplementary Material.

The selected distributions varied across domains and between sexes, reflecting differences in the distributional characteristics of CAPL-2 scores. Among girls, BCCG was selected for Physical Competence, BCPE for Daily Behavior, Motivation and Confidence, and the total CAPL-2 score, and NO for Knowledge and Understanding. Among boys, NO was selected for Physical Competence, Knowledge and Understanding, and the total CAPL-2 score; BCPE was selected for Daily Behavior; and BCCG was selected for Motivation and Confidence. All retained models achieved convergence and showed acceptable diagnostic behavior. For boys' Motivation and Confidence, although the BCPE candidate produced a lower GAIC, it showed marked residual asymmetry and kurtosis together with substantial deviations in the worm plot. The BCCG model was therefore retained because it provided acceptable residual and graphical diagnostics. Differences in the distribution families selected for girls and boys reflect statistical characteristics of the observed score distributions and should not be interpreted as evidence of underlying biological differences between sexes

### 3.3 Age- and sex-specific CAPL-2 reference percentiles.

From the selected GAMLSS models, age- and sex-specific reference percentiles were estimated for the total CAPL-2 score and its four domains. The 5th, 25th, 50th, 75th, and 95th percentiles were retained for presentation because they provide practical reference points for interpreting performance. The resulting percentile estimates are presented in Table 3.

**Table 3:** Main age- and sex-specific reference percentiles for the CAPL-2 domains and total score.

Sex	Age, years	P5	P25	P50	P75	P95
Physical competence						
Girls	8	2.91	5.97	8.81	12.27	18.36
Girls	9	3.40	6.55	9.42	12.88	18.89
Girls	10	3.92	7.15	10.04	13.47	19.39
Girls	11	4.46	7.76	10.65	14.05	19.86
Girls	12	5.04	8.38	11.27	14.62	20.31
Boys	8	3.11	8.29	11.88	15.48	20.65
Boys	9	3.54	8.98	12.77	16.55	22.00
Boys	10	3.94	9.67	13.65	17.64	23.37
Boys	11	4.32	10.35	14.54	18.73	24.76
Boys	12	4.66	11.01	15.42	19.84	26.18
Daily behavior						
Girls	8	4.05	7.26	11.22	16.17	22.64
Girls	9	4.40	7.72	11.79	16.84	23.42
Girls	10	4.76	8.19	12.35	17.51	24.17
Girls	11	5.13	8.66	12.92	18.16	24.91
Girls	12	5.52	9.15	13.49	18.81	25.63
Boys	8	6.34	11.02	16.23	21.54	26.53
Boys	9	6.27	11.03	16.32	21.71	26.78
Boys	10	6.21	11.04	16.40	21.88	27.03
Boys	11	6.15	11.04	16.49	22.05	27.28
Boys	12	6.08	11.05	16.58	22.22	27.53
Motivation and confidence						
Girls	8	16.94	20.70	24.13	27.15	29.68
Girls	9	16.44	20.27	23.76	26.81	29.36
Girls	10	15.94	19.85	23.38	26.46	29.03
Girls	11	15.44	19.43	23.01	26.12	28.71
Girls	12	14.93	19.00	22.63	25.77	28.38
Boys	8	16.38	20.90	23.69	26.27	29.71
Boys	9	16.40	20.94	23.74	26.33	29.79
Boys	10	16.42	20.98	23.79	26.40	29.87
Boys	11	16.44	21.02	23.85	26.47	29.95
Boys	12	16.45	21.06	23.90	26.53	30.00
Knowledge and understanding						
Girls	8	1.25	3.64	5.31	6.98	9.37
Girls	9	1.19	3.62	5.31	7.00	9.43
Girls	10	1.14	3.60	5.31	7.03	9.49
Girls	11	1.08	3.58	5.32	7.05	9.55
Girls	12	1.02	3.56	5.32	7.08	9.62
Boys	8	1.45	3.78	5.40	7.02	9.34
Boys	9	1.38	3.78	5.44	7.11	9.50
Boys	10	1.31	3.78	5.49	7.20	9.67
Boys	11	1.24	3.77	5.54	7.30	9.83
Boys	12	1.16	3.77	5.58	7.40	10.00
Total CAPL-2 score						
Girls	8	32.05	41.66	49.78	58.18	68.92
Girls	9	32.94	42.53	50.61	58.97	69.65
Girls	10	33.85	43.40	51.44	59.75	70.37
Girls	11	34.75	44.27	52.27	60.54	71.09
Girls	12	35.66	45.14	53.10	61.32	71.80
Boys	8	38.90	49.16	56.29	63.42	73.68
Boys	9	40.18	50.54	57.74	64.94	75.30
Boys	10	41.47	51.92	59.19	66.46	76.92
Boys	11	42.75	53.30	60.64	67.98	78.54
Boys	12	44.03	54.69	62.09	69.50	80.16

Percentiles were estimated using domain- and sex-specific GAMLSS models, with age included as a continuous explanatory variable. The main percentiles P5, P25, P50, P75, and P95 are presented.

The estimated percentile patterns varied according to age, sex, and CAPL-2 domain. Physical Competence percentiles increased progressively with age in both sexes and were generally higher among boys. Daily Behavior percentiles were also higher among boys, although age-related variation was less pronounced in this group. Motivation and Confidence showed a modest downward pattern across age among girls, whereas boys displayed comparatively stable median values across the 8–12-year age range. Knowledge and Understanding showed modest age-related variation and broadly similar percentile values between sexes. Total CAPL-2 percentiles increased with age in both sexes and were consistently higher among boys.

**Figure 1:**
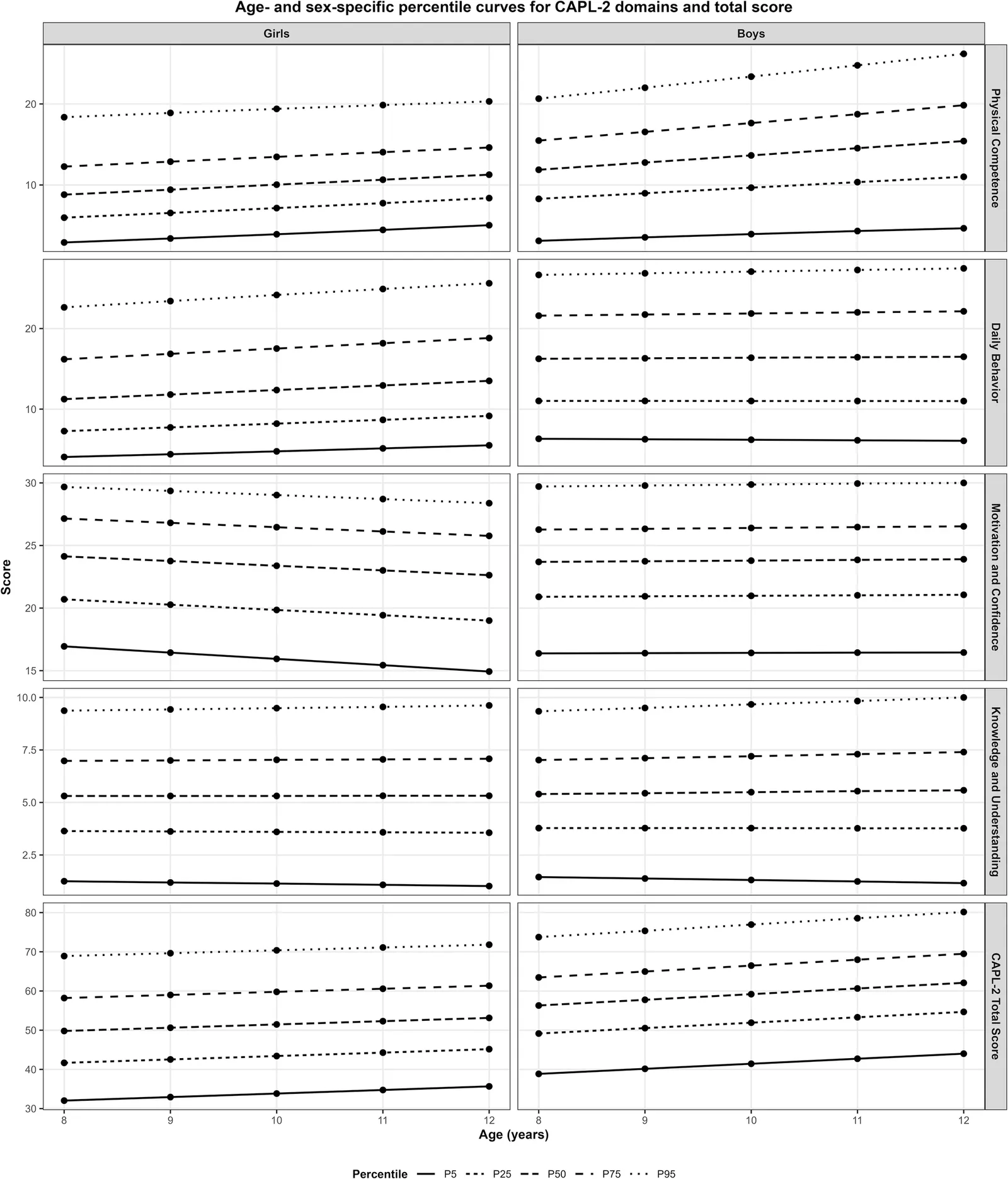
Age- and sex-specific percentile curves for the CAPL-2 domains and total score. Age- and sex-specific percentile curves (P5, P25, P50, P75, and P95) for the four CAPL-2 domains and the total CAPL-2 score in Colombian children aged 8–12 years.

Figure 1 presents the P5, P25, P50, P75, and P95 percentile curves for each CAPL-2 domain and the total score according to age and sex.

### 3.4 Correspondence between CAPL-2 interpretive categories and reference percentiles

The official CAPL-2 interpretive categories were compared with the estimated percentile distributions derived from the Colombian reference sample. For each category, the minimum score required for classification was identified, and its corresponding percentile position was estimated across age- and sex-specific distributions. As shown in Table 4, the official category thresholds occupied different relative positions across the domain-specific reference distributions.

**Table 4:** Percentile positions of the official CAPL-2 interpretive categories within the Colombian reference sample.

Domain	Official category	Median percentile	Percentile range
Total CAPL-2 score	Progressing	34.5	17.4–52.8
Total CAPL-2 score	Achieving	87.0	79.7–95.0
Total CAPL-2 score	Excelling	95.0	93.3–95.0
Physical competence	Progressing	72.8	47.4–85.1
Physical competence	Achieving	93.8	85.1–95.0
Physical competence	Excelling	95.0	93.3–95.0
Daily behavior	Progressing	26.1	15.5–48.7
Daily behavior	Achieving	82.4	78.3–93.6
Daily behavior	Excelling	95.0	93.4–95.0
Knowledge and understanding	Progressing	50.6	42.3–60.6
Knowledge and understanding	Achieving	82.0	73.5–85.4
Knowledge and understanding	Excelling	90.2	85.0–92.3
Motivation and confidence	Progressing	6.2	5.0–11.1
Motivation and confidence	Achieving	47.4	38.9–50.5
Motivation and confidence	Excelling	70.2	56.1–77.1

The median percentile represents the median of the Colombian percentile equivalents corresponding to the minimum score required for each official CAPL-2 category across all age- and sex-specific combinations. The percentile range represents the minimum and maximum values observed across those combinations. The *Beginning* category is not shown because it represents the initial classification level and does not require a minimum entry score above the lower boundary of the scale.

For the total CAPL-2 score, the progressing category corresponded to a median percentile of 34.5, whereas achieving and excelling were located at approximately the 87th and 95th percentiles, respectively. In Physical Competence, the official categories occupied particularly high positions within the reference distribution: progressing corresponded to the 72.8th percentile, achieving to the 93.8th percentile, and excelling to the 95th percentile. Similarly, the achieving category was located near the 82nd percentile for both Daily Behavior and Knowledge and Understanding.

By contrast, the official categories for Motivation and Confidence were positioned lower within the reference distribution. In this domain, progressing corresponded to approximately the 6th percentile, achieving to the 47th percentile, and excelling to the 70th percentile. These findings indicate that the relative position of the official CAPL-2 categories differed across domains. The reference percentiles therefore complement, rather than replace, the original CAPL-2 classification system by locating an individual score relative to children of the same age and sex within the Colombian reference sample.

### 3.5 Empirical percentiles for selected raw CAPL-2 components

In addition to the domain and total-score reference values, empirical percentiles were calculated by age and sex for selected raw CAPL-2 components related to Physical Competence and Daily Behavior: average daily steps, PACER laps, plank duration, and CAMSA completion time. These estimates were expressed in their original measurement units and were considered descriptive and complementary to the GAMLSS-derived reference values.

Overall, boys showed lower median CAMSA completion times and higher median values for PACER laps, average daily steps, and plank duration across most age groups. At age 12, the median PACER performance was 30 laps in boys and 18 laps in girls. Median daily step counts ranged from 9,618 to 10,880 steps/day in boys and from 6,633 to 8,520 steps/day in girls. Median plank duration was approximately 60 s or higher in boys across most age groups, whereas values in girls ranged from 44.50 to 53.00 s. The main empirical percentiles are presented in Table 5.

**Table 5:** Main empirical percentiles of the crude components of CAPL-2 by unit of measure, sex, and age.

Assessment component	Sex	Age, years	n	P5	P25	P50	P75	P95
CAMSA completion time (s)	Girls	8	84	20.00	23.15	25.56	30.09	36.34
	Girls	9	84	18.00	21.00	24.00	28.00	32.32
	Girls	10	84	18.00	22.02	24.87	28.79	33.03
	Girls	11	85	17.25	20.00	22.00	24.50	29.84
	Girls	12	88	13.56	20.00	21.25	24.27	30.96
	Boys	8	84	15.74	18.08	21.80	27.03	30.37
	Boys	9	86	12.82	18.00	21.23	25.76	34.30
	Boys	10	87	11.96	17.50	21.00	26.09	32.00
	Boys	11	85	11.62	18.00	21.00	24.00	28.14
	Boys	12	76	10.65	17.04	20.36	25.00	31.47
PACER (laps)	Girls	8	84	7.00	9.00	15.00	20.00	43.70
	Girls	9	84	7.00	11.00	17.00	28.50	46.85
	Girls	10	84	8.00	11.00	16.00	26.00	40.00
	Girls	11	85	10.00	15.00	20.00	29.00	41.60
	Girls	12	88	8.35	13.00	18.00	27.00	67.85
	Boys	8	84	7.00	11.00	19.00	24.00	47.80
	Boys	9	86	8.00	13.00	17.00	29.25	56.50
	Boys	10	87	7.30	15.00	21.00	31.00	48.70
	Boys	11	85	6.40	18.00	24.00	35.00	74.60
	Boys	12	76	10.75	22.50	30.00	50.00	64.00
Average daily steps (steps/day)	Girls	8	84	3,642	5,433	6,633	9,972	14,480
	Girls	9	84	5,195	6,228	8,375	10,640	12,545
	Girls	10	83	4,416	6,338	7,480	10,027	12,953
	Girls	11	85	4,136	6,501	7,946	11,298	14,256
	Girls	12	88	4,567	6,382	8,520	11,801	15,069
	Boys	8	83	4,966	7,139	9,618	11,967	15,699
	Boys	9	84	4,397	7,544	10,178	12,388	15,168
	Boys	10	86	4,397	7,200	10,880	13,384	16,854
	Boys	11	84	4,804	8,004	9,998	12,884	16,415
	Boys	12	74	4,231	7,892	10,320	12,991	16,317
Plank duration (s)	Girls	8	84	15.77	29.56	44.50	67.72	221.00
	Girls	9	84	23.85	33.97	44.50	58.00	105.95
	Girls	10	84	23.24	38.97	52.00	68.97	91.70
	Girls	11	85	19.20	41.16	53.00	73.90	147.00
	Girls	12	88	20.35	36.75	49.59	69.93	106.94
	Boys	8	84	23.15	41.31	60.00	88.52	174.28
	Boys	9	86	23.24	42.00	59.00	76.17	140.75
	Boys	10	87	27.69	47.00	60.00	84.94	177.90
	Boys	11	85	18.23	39.00	60.00	92.22	153.20
	Boys	12	76	16.51	47.00	63.05	85.88	130.06

Percentiles were calculated empirically from the observed data for each assessment component, sex, and age combination. For CAMSA, the shortest completion time across the two attempts was used; therefore, lower values indicate better performance. For PACER, average daily steps, and plank duration, higher values indicate better performance.

## 4 Discussion

This study developed age- and sex-specific reference values for the CAPL-2 total score and its four domains in a geographically diverse multicenter sample of Colombian schoolchildren aged 8–12 years. Using GAMLSS, the analysis generated smoothed percentile distributions that account for age-related variation and sex-specific differences in CAPL-2 scores. These reference values provide a contextual framework for interpreting physical literacy performance by allowing an individual score to be located relative to children of the same age and sex within the Colombian reference sample. Importantly, these percentile references are intended to complement, rather than replace, the original CAPL-2 interpretive categories.

One of the clearest patterns observed in the reference distributions was the progressive increase in Physical Competence and total CAPL-2 scores across age, particularly among boys. Similar age- and sex-related patterns have been reported in previous CAPL-2 studies conducted in other populations. The Chinese cross-validation of the CAPL-2 identified sex differences in total physical literacy, with boys generally achieving higher scores than girls (5), while studies conducted in Danish and Pakistani schoolchildren have also reported variation in CAPL-2 performance according to age and sex (6, 10). In the Pakistani adaptation, Physical Competence increased with age and boys generally obtained higher scores across several CAPL-2 domains (10). However, the sex differences observed in the present study should not be interpreted as reflecting biological factors alone. They may also be influenced by differences in maturation, participation in organized sport and physical activity, opportunities for movement practice, and gender-related sociocultural conditions. Because these factors were not directly measured in the present study, they should be considered plausible contextual explanations rather than established mechanisms. Taken together, these findings support the relevance of considering age and sex when interpreting CAPL-2 performance and reinforce the usefulness of age- and sex-specific reference distributions rather than a single undifferentiated reference value.

A different pattern was observed for Motivation and Confidence. Girls showed a modest decrease in median scores across the age range examined, whereas boys displayed comparatively stable median values. Similar sex-related patterns have been described in previous CAPL-2 research. In the Pakistani CAPL-2 study, girls showed decreasing Motivation and Confidence scores with increasing age, whereas boys generally maintained higher scores (10). However, because the present study was cross-sectional, these differences should not be interpreted as individual developmental trajectories. Rather, they represent age-group differences within the reference sample. The observed pattern may reflect a combination of individual, social, and contextual influences on motivation and confidence, but these potential mechanisms were not directly assessed. This distinction is relevant because physical literacy is multidimensional, and higher performance in physical or behavioral components does not necessarily imply parallel differences in motivation or confidence. Sex-related differences were also evident in Daily Behavior and in several of the raw physical assessment components. Boys generally showed higher Daily Behavior scores and higher empirical medians for average daily steps, PACER laps, and plank duration, while also recording shorter CAMSA completion times across most age groups. Comparable patterns have been reported in Canadian children, in whom boys accumulated more daily steps than girls and achieved higher scores in several physical competence measures, including PACER, plank, and CAMSA (21). These findings are also consistent with CAPL-2 research from other cultural settings reporting generally higher physical literacy performance among boys (5, 10). Nevertheless, the present data do not allow these differences to be attributed to biological sex alone. Differences in habitual physical activity, participation in sport, opportunities for movement practice, school and community environments, and other sociocultural factors may also contribute. Accordingly, sex-specific reference values are useful for descriptive interpretation of CAPL-2 performance, but the observed differences should not be regarded as evidence of fixed or inherently biological performance gaps. Knowledge and understanding showed a comparatively stable pattern across age, with only modest differences between girls and boys. This contrasts with the clearer age- and sex-related variation observed in the physical and behavioral domains. Previous cross-cultural evaluations of CAPL-2 have shown that the cognitive domain may present measurement characteristics that differ across settings. In the Chinese cross-validation study, one Knowledge and Understanding item was removed to improve model fit (5), whereas the Danish adaptation retained the original indicators within a satisfactory four-domain structure (6). These findings highlight the importance of interpreting the cognitive dimension of physical literacy within the cultural and educational context in which CAPL-2 is administered.

A particularly relevant finding was the variable percentile position of the official CAPL-2 interpretive categories within the Colombian reference distributions. The CAPL-2 classification system was originally designed to categorize performance as Beginning, Progressing, Achieving, or Excelling using age- and sex-specific normative thresholds derived from Canadian data (3). In the present study, however, the minimum scores required to enter these categories occupied markedly different relative positions across domains. For example, the threshold for Achieving corresponded to approximately the 94th percentile in Physical Competence and the 82nd percentile in both Daily Behavior and Knowledge and Understanding, whereas in Motivation and Confidence it was located near the 47th percentile. These differences indicate that identical official category labels may represent substantially different relative positions within the Colombian reference distributions depending on the domain considered.

This finding should not be interpreted as evidence that the original CAPL-2 thresholds are inappropriate or should be replaced. Rather, the two approaches provide different but complementary information. The original CAPL-2 categories preserve comparability with the standardized interpretation system developed for the instrument (3), whereas the age- and sex-specific percentiles derived in this study indicate a child's relative position within the Colombian reference sample. Reporting both forms of interpretation may therefore provide a more informative characterization of CAPL-2 performance than either approach used in isolation.

The use of GAMLSS represents an important methodological strength of the present study. Unlike approaches based exclusively on empirical percentiles or a single distributional assumption, GAMLSS provides a flexible framework in which parameters describing location, scale, skewness, and kurtosis can vary as functions of explanatory variables such as age (14, 15, 18). This approach has been applied extensively in the construction of pediatric reference distributions, including international child growth standards and age- and sex-specific physical fitness reference values (22, 23). In the present study, candidate distributions were evaluated not only according to information criteria but also through convergence assessment, normalized quantile residuals, and worm plots. This diagnostic approach was particularly relevant when alternative distributions yielded lower GAIC values but showed inadequate residual behavior. The final selected distributions therefore reflected a balance between statistical fit and diagnostic adequacy rather than reliance on a single model-selection criterion. Nevertheless, these reference values should be interpreted within the conceptual boundaries of the instrument. Dudley and Cairney have questioned the content validity of the CAPL-2, arguing that its operational components may not fully represent the conceptual breadth of physical literacy (24). Accordingly, the percentiles developed in this study should be understood as reference values for CAPL-2 performance within the Colombian reference sample, rather than as absolute reference standards for the broader construct of physical literacy.

Several strengths should be highlighted. First, the study included 843 schoolchildren from the six geographical subregions of Colombia, with a balanced distribution by sex and age. Second, data completeness was high across all CAPL-2 outcomes, exceeding 96%, which minimized the potential impact of missing information on percentile estimation. Third, the use of domain- and sex-specific GAMLSS models allowed the reference distributions to accommodate differences in location, variability, skewness, and kurtosis rather than relying on a single distributional assumption, consistent with flexible approaches previously used for the construction of pediatric reference curves (25). Candidate models were also subjected to residual and graphical diagnostic assessment, which reduced the risk of selecting a distribution solely on the basis of information criteria. Finally, the presentation of both GAMLSS-derived percentiles for CAPL-2 domains and total score and empirical percentiles for selected raw assessment components provides complementary levels of interpretation while preserving the original CAPL-2 scoring framework. Several limitations should also be considered. Although participants were recruited from all six geographical subregions of Colombia and the sample was balanced by age and sex, the participating cities and educational institutions were selected purposively rather than through probability-based sampling. Consequently, the resulting percentiles should be interpreted as reference values derived from a geographically diverse multicenter Colombian sample rather than as nationally representative population norms. The sample was drawn from public educational institutions, and rural or remote settings, private schools, socioeconomic conditions, and school-level characteristics were not specifically modeled; therefore, the applicability of these reference values to those populations and contexts should be interpreted cautiously. In addition, the clustered structure of the data by school, city, and geographical subregion was not incorporated into the GAMLSS models, which may have resulted in underestimated uncertainty around some percentile estimates.

The cross-sectional design describes age-group differences in score distributions but does not allow conclusions regarding individual developmental trajectories or changes over time. External validation in an independent Colombian sample was not performed. Finally, although the overall sample was relatively large, empirical percentiles for individual assessment components were calculated within age- and sex-specific subgroups; therefore, estimates at the extreme percentiles should be interpreted with greater caution because they are based on smaller numbers of observations. Future studies using probability-based sampling, broader representation of rural and urban settings, public and private schools, different socioeconomic contexts, multilevel or cluster-aware modeling approaches, longitudinal designs, and independent external validation would help evaluate the generalizability and stability of these reference distributions. From an applied perspective, the reference values generated in this study may support a more contextualized interpretation of CAPL-2 performance in Colombian schoolchildren. Their use may be particularly useful in research, school-based assessment, and physical activity promotion initiatives by allowing domain and total scores to be interpreted relative to children of the same age and sex within the reference sample. However, these percentiles should not be used as diagnostic thresholds or as substitutes for the original CAPL-2 categories. Future studies should evaluate the stability of these reference distributions in independent Colombian samples, including rural and urban settings, public and private schools, and different socioeconomic contexts. Additional research should also examine longitudinal changes in CAPL-2 performance and evaluate whether individual changes over time are associated with physical activity, motor competence, health-related fitness, mental and emotional well-being, and other relevant outcomes.

## Conclusions

This study developed age- and sex-specific reference values for the CAPL-2 total score and its four domains in a geographically diverse multicenter sample of Colombian schoolchildren aged 8–12 years. The GAMLSS-derived percentile distributions revealed distinct age- and sex-related patterns across CAPL-2 domains and provided a complementary framework for interpreting children's scores relative to peers of the same age and sex within the reference sample. The correspondence analysis further showed that the original CAPL-2 interpretive categories occupied different relative percentile positions across domains, supporting the combined use of the original classification system and contextual percentile information. These reference values should not be interpreted as diagnostic thresholds, developmental trajectories, or nationally representative population norms. Rather, they provide a contextual tool for research and school-based assessment of CAPL-2 performance in Colombian children and should be externally validated in broader and independently sampled populations.

## Data Availability

The raw data supporting the conclusions of this article will be made available by the authors, without undue reservation.
