# Supplementary material for: Age- and sex-specific reference values for physical literacy in Colombian children: a GAMLSS analysis of the CAPL-2
Source: Front Sports Act Living. 2026 Sep 7;8:1963012. doi: 10.3389/fspor.2026.1963012 (PMC13590728; doi:10.3389/fspor.2026.1963012)
Supplement: Supplementary file 1 [file Table1.docx]

**Supplementary Table S1. Complete CAPL-2 reference percentiles by domain, sex, and age in the Colombian reference sample**

| **Domain** | **Sex** | **Model** | **Age** | **P5** | **P10** | **P15** | **P20** | **P25** | **P30** | **P35** | **P40** | **P45** | **P50** | **P55** | **P60** | **P65** | **P70** | **P75** | **P80** | **P85** | **P90** | **P95** |
| --- | --- | --- | --- | --- | --- | --- | --- | --- | --- | --- | --- | --- | --- | --- | --- | --- | --- | --- | --- | --- | --- | --- |
| Physical Competence | Girls | BCCG | 8 | 2.91 | 3.92 | 4.69 | 5.36 | 5.97 | 6.55 | 7.11 | 7.67 | 8.23 | 8.81 | 9.41 | 10.04 | 10.71 | 11.44 | 12.27 | 13.22 | 14.39 | 15.92 | 18.36 |
| Physical Competence | Girls | BCCG | 9 | 3.40 | 4.45 | 5.24 | 5.93 | 6.55 | 7.14 | 7.71 | 8.28 | 8.85 | 9.42 | 10.02 | 10.65 | 11.32 | 12.06 | 12.88 | 13.82 | 14.97 | 16.49 | 18.89 |
| Physical Competence | Girls | BCCG | 10 | 3.92 | 5.00 | 5.82 | 6.52 | 7.15 | 7.75 | 8.32 | 8.89 | 9.46 | 10.04 | 10.64 | 11.26 | 11.93 | 12.66 | 13.47 | 14.41 | 15.54 | 17.03 | 19.39 |
| Physical Competence | Girls | BCCG | 11 | 4.46 | 5.58 | 6.41 | 7.12 | 7.76 | 8.36 | 8.94 | 9.51 | 10.08 | 10.65 | 11.25 | 11.87 | 12.53 | 13.25 | 14.05 | 14.97 | 16.09 | 17.56 | 19.86 |
| Physical Competence | Girls | BCCG | 12 | 5.04 | 6.18 | 7.03 | 7.74 | 8.38 | 8.98 | 9.56 | 10.13 | 10.69 | 11.27 | 11.86 | 12.47 | 13.13 | 13.83 | 14.62 | 15.53 | 16.62 | 18.06 | 20.31 |
| Physical Competence | Boys | NO | 8 | 3.11 | 5.05 | 6.36 | 7.40 | 8.29 | 9.09 | 9.83 | 10.53 | 11.21 | 11.88 | 12.55 | 13.23 | 13.94 | 14.68 | 15.48 | 16.37 | 17.41 | 18.72 | 20.65 |
| Physical Competence | Boys | NO | 9 | 3.54 | 5.58 | 6.95 | 8.05 | 8.98 | 9.83 | 10.61 | 11.35 | 12.06 | 12.77 | 13.47 | 14.19 | 14.93 | 15.71 | 16.55 | 17.49 | 18.58 | 19.96 | 22.00 |
| Physical Competence | Boys | NO | 10 | 3.94 | 6.09 | 7.53 | 8.68 | 9.67 | 10.56 | 11.38 | 12.16 | 12.91 | 13.65 | 14.40 | 15.15 | 15.93 | 16.75 | 17.64 | 18.62 | 19.77 | 21.22 | 23.37 |
| Physical Competence | Boys | NO | 11 | 4.32 | 6.57 | 8.10 | 9.31 | 10.35 | 11.28 | 12.14 | 12.96 | 13.76 | 14.54 | 15.32 | 16.11 | 16.93 | 17.80 | 18.73 | 19.77 | 20.98 | 22.50 | 24.76 |
| Physical Competence | Boys | NO | 12 | 4.66 | 7.04 | 8.64 | 9.92 | 11.01 | 11.99 | 12.90 | 13.77 | 14.60 | 15.42 | 16.25 | 17.08 | 17.94 | 18.85 | 19.84 | 20.93 | 22.20 | 23.81 | 26.18 |
| Daily Behavior | Girls | BCPE | 8 | 4.05 | 5.00 | 5.79 | 6.53 | 7.26 | 7.99 | 8.75 | 9.53 | 10.36 | 11.22 | 12.12 | 13.05 | 14.04 | 15.07 | 16.17 | 17.37 | 18.72 | 20.35 | 22.64 |
| Daily Behavior | Girls | BCPE | 9 | 4.40 | 5.39 | 6.21 | 6.97 | 7.72 | 8.47 | 9.25 | 10.06 | 10.90 | 11.79 | 12.70 | 13.66 | 14.67 | 15.72 | 16.84 | 18.06 | 19.44 | 21.10 | 23.42 |
| Daily Behavior | Girls | BCPE | 10 | 4.76 | 5.78 | 6.63 | 7.42 | 8.19 | 8.96 | 9.76 | 10.59 | 11.45 | 12.35 | 13.29 | 14.27 | 15.29 | 16.37 | 17.51 | 18.75 | 20.14 | 21.83 | 24.17 |
| Daily Behavior | Girls | BCPE | 11 | 5.13 | 6.19 | 7.06 | 7.87 | 8.66 | 9.46 | 10.28 | 11.12 | 12.00 | 12.92 | 13.88 | 14.87 | 15.91 | 17.00 | 18.16 | 19.42 | 20.83 | 22.54 | 24.91 |
| Daily Behavior | Girls | BCPE | 12 | 5.52 | 6.61 | 7.50 | 8.33 | 9.15 | 9.96 | 10.80 | 11.66 | 12.56 | 13.49 | 14.46 | 15.47 | 16.53 | 17.63 | 18.81 | 20.08 | 21.51 | 23.23 | 25.63 |
| Daily Behavior | Boys | BCPE | 8 | 6.34 | 7.74 | 8.90 | 9.98 | 11.02 | 12.06 | 13.09 | 14.13 | 15.18 | 16.23 | 17.28 | 18.34 | 19.40 | 20.47 | 21.54 | 22.64 | 23.77 | 25.01 | 26.53 |
| Daily Behavior | Boys | BCPE | 9 | 6.27 | 7.70 | 8.88 | 9.97 | 11.03 | 12.08 | 13.13 | 14.19 | 15.25 | 16.32 | 17.38 | 18.46 | 19.54 | 20.62 | 21.71 | 22.82 | 23.98 | 25.24 | 26.78 |
| Daily Behavior | Boys | BCPE | 10 | 6.21 | 7.66 | 8.85 | 9.96 | 11.04 | 12.10 | 13.17 | 14.24 | 15.32 | 16.40 | 17.49 | 18.58 | 19.67 | 20.77 | 21.88 | 23.01 | 24.18 | 25.46 | 27.03 |
| Daily Behavior | Boys | BCPE | 11 | 6.15 | 7.61 | 8.82 | 9.95 | 11.04 | 12.13 | 13.21 | 14.30 | 15.39 | 16.49 | 17.59 | 18.70 | 19.81 | 20.92 | 22.05 | 23.20 | 24.39 | 25.69 | 27.28 |
| Daily Behavior | Boys | BCPE | 12 | 6.08 | 7.57 | 8.80 | 9.94 | 11.05 | 12.15 | 13.25 | 14.35 | 15.46 | 16.58 | 17.69 | 18.82 | 19.95 | 21.08 | 22.22 | 23.39 | 24.60 | 25.91 | 27.53 |
| Motivation and Confidence | Girls | BCPE | 8 | 16.94 | 18.16 | 19.10 | 19.93 | 20.70 | 21.43 | 22.14 | 22.82 | 23.48 | 24.13 | 24.76 | 25.38 | 25.98 | 26.57 | 27.15 | 27.73 | 28.32 | 28.94 | 29.68 |
| Motivation and Confidence | Girls | BCPE | 9 | 16.44 | 17.69 | 18.65 | 19.49 | 20.27 | 21.02 | 21.73 | 22.43 | 23.10 | 23.76 | 24.40 | 25.02 | 25.63 | 26.22 | 26.81 | 27.39 | 27.98 | 28.61 | 29.36 |
| Motivation and Confidence | Girls | BCPE | 10 | 15.94 | 17.21 | 18.19 | 19.05 | 19.85 | 20.61 | 21.33 | 22.04 | 22.72 | 23.38 | 24.03 | 24.66 | 25.27 | 25.87 | 26.46 | 27.05 | 27.65 | 28.28 | 29.03 |
| Motivation and Confidence | Girls | BCPE | 11 | 15.44 | 16.74 | 17.74 | 18.62 | 19.43 | 20.19 | 20.93 | 21.64 | 22.34 | 23.01 | 23.66 | 24.29 | 24.91 | 25.52 | 26.12 | 26.71 | 27.31 | 27.95 | 28.71 |
| Motivation and Confidence | Girls | BCPE | 12 | 14.93 | 16.27 | 17.29 | 18.18 | 19.00 | 19.78 | 20.53 | 21.25 | 21.95 | 22.63 | 23.29 | 23.93 | 24.56 | 25.17 | 25.77 | 26.37 | 26.97 | 27.61 | 28.38 |
| Motivation and Confidence | Boys | BCCG | 8 | 16.38 | 18.16 | 19.30 | 20.17 | 20.90 | 21.54 | 22.12 | 22.67 | 23.18 | 23.69 | 24.18 | 24.68 | 25.18 | 25.71 | 26.27 | 26.88 | 27.59 | 28.45 | 29.71 |
| Motivation and Confidence | Boys | BCCG | 9 | 16.40 | 18.19 | 19.33 | 20.21 | 20.94 | 21.58 | 22.17 | 22.72 | 23.24 | 23.74 | 24.24 | 24.74 | 25.24 | 25.77 | 26.33 | 26.95 | 27.66 | 28.53 | 29.79 |
| Motivation and Confidence | Boys | BCCG | 10 | 16.42 | 18.21 | 19.36 | 20.25 | 20.98 | 21.63 | 22.22 | 22.77 | 23.29 | 23.79 | 24.29 | 24.79 | 25.31 | 25.84 | 26.40 | 27.02 | 27.73 | 28.61 | 29.87 |
| Motivation and Confidence | Boys | BCCG | 11 | 16.44 | 18.24 | 19.40 | 20.28 | 21.02 | 21.67 | 22.26 | 22.82 | 23.34 | 23.85 | 24.35 | 24.85 | 25.37 | 25.90 | 26.47 | 27.09 | 27.80 | 28.68 | 29.95 |
| Motivation and Confidence | Boys | BCCG | 12 | 16.45 | 18.27 | 19.43 | 20.32 | 21.06 | 21.72 | 22.31 | 22.86 | 23.39 | 23.90 | 24.41 | 24.91 | 25.43 | 25.96 | 26.53 | 27.16 | 27.87 | 28.76 | 30.00 |
| Knowledge and Understanding | Girls | NO | 8 | 1.25 | 2.14 | 2.75 | 3.23 | 3.64 | 4.01 | 4.36 | 4.68 | 5.00 | 5.31 | 5.62 | 5.93 | 6.26 | 6.60 | 6.98 | 7.39 | 7.87 | 8.47 | 9.37 |
| Knowledge and Understanding | Girls | NO | 9 | 1.19 | 2.10 | 2.72 | 3.20 | 3.62 | 4.00 | 4.35 | 4.68 | 5.00 | 5.31 | 5.63 | 5.95 | 6.28 | 6.63 | 7.00 | 7.42 | 7.91 | 8.52 | 9.43 |
| Knowledge and Understanding | Girls | NO | 10 | 1.14 | 2.06 | 2.68 | 3.18 | 3.60 | 3.98 | 4.34 | 4.67 | 5.00 | 5.31 | 5.63 | 5.96 | 6.29 | 6.65 | 7.03 | 7.45 | 7.95 | 8.57 | 9.49 |
| Knowledge and Understanding | Girls | NO | 11 | 1.08 | 2.02 | 2.65 | 3.15 | 3.58 | 3.97 | 4.32 | 4.66 | 4.99 | 5.32 | 5.64 | 5.97 | 6.31 | 6.67 | 7.05 | 7.49 | 7.99 | 8.62 | 9.55 |
| Knowledge and Understanding | Girls | NO | 12 | 1.02 | 1.97 | 2.61 | 3.12 | 3.56 | 3.95 | 4.31 | 4.66 | 4.99 | 5.32 | 5.65 | 5.98 | 6.33 | 6.69 | 7.08 | 7.52 | 8.03 | 8.67 | 9.62 |
| Knowledge and Understanding | Boys | NO | 8 | 1.45 | 2.32 | 2.91 | 3.38 | 3.78 | 4.14 | 4.47 | 4.79 | 5.10 | 5.40 | 5.70 | 6.01 | 6.32 | 6.66 | 7.02 | 7.42 | 7.88 | 8.47 | 9.34 |
| Knowledge and Understanding | Boys | NO | 9 | 1.38 | 2.28 | 2.89 | 3.37 | 3.78 | 4.15 | 4.49 | 4.82 | 5.13 | 5.44 | 5.75 | 6.07 | 6.39 | 6.74 | 7.11 | 7.52 | 8.00 | 8.61 | 9.50 |
| Knowledge and Understanding | Boys | NO | 10 | 1.31 | 2.23 | 2.86 | 3.35 | 3.78 | 4.16 | 4.51 | 4.85 | 5.17 | 5.49 | 5.81 | 6.13 | 6.47 | 6.82 | 7.20 | 7.63 | 8.12 | 8.75 | 9.67 |
| Knowledge and Understanding | Boys | NO | 11 | 1.24 | 2.19 | 2.83 | 3.34 | 3.77 | 4.17 | 4.53 | 4.87 | 5.21 | 5.54 | 5.86 | 6.20 | 6.54 | 6.91 | 7.30 | 7.74 | 8.24 | 8.89 | 9.83 |
| Knowledge and Understanding | Boys | NO | 12 | 1.16 | 2.14 | 2.80 | 3.32 | 3.77 | 4.17 | 4.55 | 4.90 | 5.24 | 5.58 | 5.92 | 6.26 | 6.62 | 6.99 | 7.40 | 7.85 | 8.37 | 9.03 | 10.00 |
| CAPL-2 total score | Girls | BCPE | 8 | 32.05 | 35.40 | 37.82 | 39.84 | 41.66 | 43.37 | 45.00 | 46.60 | 48.19 | 49.78 | 51.39 | 53.01 | 54.66 | 56.37 | 58.18 | 60.14 | 62.37 | 65.07 | 68.92 |
| CAPL-2 total score | Girls | BCPE | 9 | 32.94 | 36.28 | 38.69 | 40.72 | 42.53 | 44.23 | 45.85 | 47.45 | 49.03 | 50.61 | 52.21 | 53.82 | 55.47 | 57.17 | 58.97 | 60.92 | 63.13 | 65.82 | 69.65 |
| CAPL-2 total score | Girls | BCPE | 10 | 33.85 | 37.17 | 39.58 | 41.59 | 43.40 | 45.08 | 46.71 | 48.29 | 49.87 | 51.44 | 53.03 | 54.63 | 56.27 | 57.97 | 59.75 | 61.70 | 63.89 | 66.57 | 70.37 |
| CAPL-2 total score | Girls | BCPE | 11 | 34.75 | 38.07 | 40.46 | 42.47 | 44.27 | 45.95 | 47.56 | 49.14 | 50.71 | 52.27 | 53.85 | 55.45 | 57.08 | 58.76 | 60.54 | 62.47 | 64.65 | 67.31 | 71.09 |
| CAPL-2 total score | Girls | BCPE | 12 | 35.66 | 38.97 | 41.35 | 43.35 | 45.14 | 46.81 | 48.41 | 49.99 | 51.54 | 53.10 | 54.67 | 56.26 | 57.88 | 59.55 | 61.32 | 63.24 | 65.41 | 68.05 | 71.80 |
| CAPL-2 total score | Boys | NO | 8 | 38.90 | 42.74 | 45.33 | 47.39 | 49.16 | 50.74 | 52.21 | 53.61 | 54.96 | 56.29 | 57.62 | 58.97 | 60.36 | 61.83 | 63.42 | 65.19 | 67.25 | 69.84 | 73.68 |
| CAPL-2 total score | Boys | NO | 9 | 40.18 | 44.06 | 46.68 | 48.76 | 50.54 | 52.14 | 53.63 | 55.04 | 56.40 | 57.74 | 59.08 | 60.44 | 61.85 | 63.34 | 64.94 | 66.72 | 68.80 | 71.42 | 75.30 |
| CAPL-2 total score | Boys | NO | 10 | 41.47 | 45.38 | 48.02 | 50.12 | 51.92 | 53.54 | 55.04 | 56.46 | 57.84 | 59.19 | 60.55 | 61.92 | 63.34 | 64.84 | 66.46 | 68.26 | 70.36 | 73.00 | 76.92 |
| CAPL-2 total score | Boys | NO | 11 | 42.75 | 46.70 | 49.37 | 51.49 | 53.30 | 54.94 | 56.45 | 57.89 | 59.28 | 60.64 | 62.01 | 63.40 | 64.83 | 66.35 | 67.98 | 69.80 | 71.92 | 74.59 | 78.54 |
| CAPL-2 total score | Boys | NO | 12 | 44.03 | 48.02 | 50.71 | 52.85 | 54.69 | 56.33 | 57.86 | 59.31 | 60.71 | 62.09 | 63.47 | 64.88 | 66.33 | 67.85 | 69.50 | 71.34 | 73.48 | 76.17 | 80.16 |

**Note.** Percentiles were estimated using domain- and sex-specific generalized additive models for location, scale, and shape (GAMLSS), with age included as a continuous explanatory variable. The table presents percentiles from P5 to P95 in five-percentile increments. Scores should be interpreted within the theoretical limits of each domain: Physical Competence, Daily Behavior, and Motivation and Confidence, 0–30 points; Knowledge and Understanding, 0–10 points; and CAPL-2 total score, 0–100 points. Values marginally exceeding the possible limits of the instrument were restricted to the corresponding theoretical range. NO, normal distribution; BCCG, Box-Cox Cole and Green; BCPE, Box-Cox power exponential; CAPL-2, Canadian Assessment of Physical Literacy, second edition.
