# Supplementary material for: Age- and sex-specific reference values for physical literacy in Colombian children: a GAMLSS analysis of the CAPL-2
Source: Front Sports Act Living. 2026 Sep 7;8:1963012. doi: 10.3389/fspor.2026.1963012 (PMC13590728; doi:10.3389/fspor.2026.1963012)
Supplement: Supplementary file 2 [file Table2.docx]

**Supplementary Table S2. Comparison of GAMLSS models for estimating CAPL-2 percentiles in the Colombian reference sample**

| **Domain** | **Variable** | **Sex** | **n** | **Model** | **Converged** | **GAIC** | **SBC** | **Global deviance** | **Observation** | **GAIC rank** |
| --- | --- | --- | --- | --- | --- | --- | --- | --- | --- | --- |
| Physical Competence | pc_score | Girls | 417 | BCCG | Yes | 2452.32 | 2472.48 | 2442.32 | — | 1 |
| Physical Competence | pc_score | Girls | 417 | BCPE | Yes | 2453.62 | 2477.82 | 2441.62 | — | 2 |
| Physical Competence | pc_score | Girls | 417 | BCT | Yes | 2454.32 | 2478.52 | 2442.32 | — | 3 |
| Physical Competence | pc_score | Girls | 417 | NO | Yes | 2488.03 | 2504.16 | 2480.03 | — | 4 |
| Physical Competence | pc_score | Boys | 412 | NO | Yes | 2638.96 | 2655.04 | 2630.96 | — | 1 |
| Physical Competence | pc_score | Boys | 412 | BCCG | No | — | — | — | Response variable out of range | 2 |
| Physical Competence | pc_score | Boys | 412 | BCPE | No | — | — | — | Response variable out of range | 3 |
| Physical Competence | pc_score | Boys | 412 | BCT | No | — | — | — | Response variable out of range | 4 |
| Daily Behavior | db_score | Girls | 414 | BCPE | Yes | 2613.88 | 2638.03 | 2601.88 | — | 1 |
| Daily Behavior | db_score | Girls | 414 | BCCG | Yes | 2636.27 | 2656.40 | 2626.27 | — | 2 |
| Daily Behavior | db_score | Girls | 414 | BCT | Yes | 2638.27 | 2662.42 | 2626.27 | — | 3 |
| Daily Behavior | db_score | Girls | 414 | NO | Yes | 2688.09 | 2704.19 | 2680.09 | — | 4 |
| Daily Behavior | db_score | Boys | 405 | BCPE | Yes | 2637.97 | 2661.99 | 2625.97 | — | 1 |
| Daily Behavior | db_score | Boys | 405 | BCCG | Yes | 2668.56 | 2688.58 | 2658.56 | — | 2 |
| Daily Behavior | db_score | Boys | 405 | BCT | Yes | 2670.73 | 2694.75 | 2658.73 | — | 3 |
| Daily Behavior | db_score | Boys | 405 | NO | Yes | 2679.39 | 2695.40 | 2671.39 | — | 4 |
| Motivation and Confidence | mc_score | Girls | 419 | BCPE | Yes | 2341.18 | 2365.41 | 2329.18 | — | 1 |
| Motivation and Confidence | mc_score | Girls | 419 | NO | Yes | 2386.63 | 2402.79 | 2378.63 | — | 2 |
| Motivation and Confidence | mc_score | Girls | 419 | BCCG | Yes | 2386.80 | 2406.99 | 2376.80 | — | 3 |
| Motivation and Confidence | mc_score | Girls | 419 | BCT | Yes | 2388.80 | 2413.03 | 2376.80 | — | 4 |
| Motivation and Confidence | mc_score | Boys | 415 | BCPE | Yes | 2269.69 | 2293.86 | 2257.69 | Not selected: inadequate normalized quantile residuals and worm-plot diagnostics | 1 |
| Motivation and Confidence | mc_score | Boys | 415 | BCT | No | — | — | — | RS algorithm did not converge after 500 iterations | — |
| Motivation and Confidence | mc_score | Boys | 415 | BCCG | Yes | 2352.31 | 2372.45 | 2342.31 | Selected final model based on acceptable residual and worm-plot diagnostics | 2 |
| Motivation and Confidence | mc_score | Boys | 415 | NO | Yes | 2355.60 | 2371.72 | 2347.60 | Not selected | 3 |
| Knowledge and Understanding | ku_score | Girls | 423 | NO | Yes | 1997.36 | 2013.55 | 1989.36 | — | 1 |
| Knowledge and Understanding | ku_score | Girls | 423 | BCCG | No | — | — | — | Response variable out of range | 2 |
| Knowledge and Understanding | ku_score | Girls | 423 | BCPE | No | — | — | — | Response variable out of range | 3 |
| Knowledge and Understanding | ku_score | Girls | 423 | BCT | No | — | — | — | Response variable out of range | 4 |
| Knowledge and Understanding | ku_score | Boys | 416 | NO | Yes | 1963.13 | 1979.25 | 1955.13 | — | 1 |
| Knowledge and Understanding | ku_score | Boys | 416 | BCCG | No | — | — | — | Response variable out of range | 2 |
| Knowledge and Understanding | ku_score | Boys | 416 | BCPE | No | — | — | — | Response variable out of range | 3 |
| Knowledge and Understanding | ku_score | Boys | 416 | BCT | No | — | — | — | Response variable out of range | 4 |
| CAPL-2 Total Score | capl_score | Girls | 413 | BCPE | Yes | 3174.30 | 3198.44 | 3162.30 | — | 1 |
| CAPL-2 Total Score | capl_score | Girls | 413 | NO | Yes | 3175.47 | 3191.57 | 3167.47 | — | 2 |
| CAPL-2 Total Score | capl_score | Girls | 413 | BCCG | Yes | 3176.60 | 3196.72 | 3166.60 | — | 3 |
| CAPL-2 Total Score | capl_score | Girls | 413 | BCT | Yes | 3178.60 | 3202.74 | 3166.60 | — | 4 |
| CAPL-2 Total Score | capl_score | Boys | 403 | NO | Yes | 3067.32 | 3083.32 | 3059.32 | — | 1 |
| CAPL-2 Total Score | capl_score | Boys | 403 | BCPE | Yes | 3067.60 | 3091.60 | 3055.60 | — | 2 |
| CAPL-2 Total Score | capl_score | Boys | 403 | BCCG | Yes | 3068.40 | 3088.39 | 3058.40 | — | 3 |
| CAPL-2 Total Score | capl_score | Boys | 403 | BCT | Yes | 3070.40 | 3094.39 | 3058.40 | — | 4 |

Note. NO: normal distribution; BCCG: Box-Cox Cole and Green; BCPE: Box-Cox Power Exponential; BCT: Box-Cox t; GAMLSS: generalized additive models for location, scale, and shape; GAIC: generalized Akaike information criterion; SBC: Schwarz Bayesian criterion; CAPL-2: Canadian Assessment of Physical Literacy, second edition. GAIC rank orders candidate models from the lowest to the highest GAIC value within each domain-by-sex combination. For models that did not converge, GAIC, SBC, and global deviance values are not reported. The observation "Response variable out of range" corresponds to the message generated during model fitting. For boys’ Motivation and Confidence, the BCPE model yielded the lowest GAIC but was not retained because normalized quantile residuals and worm-plot diagnostics indicated inadequate fit. The BCCG model was therefore selected as the final model based on convergence and acceptable diagnostic performance.
