# Supplementary material for: Age- and sex-specific reference values for physical literacy in Colombian children: a GAMLSS analysis of the CAPL-2
Source: Front Sports Act Living. 2026 Sep 7;8:1963012. doi: 10.3389/fspor.2026.1963012 (PMC13590728; doi:10.3389/fspor.2026.1963012)
Supplement: Supplementary file 3 [file Table3.docx]

**Supplementary Table S3. Complete empirical percentiles for selected raw CAPL-2 components by sex and age**

| **Component** | **Variable** | **Unit** | **Sex** | **Age** | **n** | **P5** | **P10** | **P15** | **P20** | **P25** | **P30** | **P35** | **P40** | **P45** | **P50** | **P55** | **P60** | **P65** | **P70** | **P75** | **P80** | **P85** | **P90** | **P95** |
| --- | --- | --- | --- | --- | --- | --- | --- | --- | --- | --- | --- | --- | --- | --- | --- | --- | --- | --- | --- | --- | --- | --- | --- | --- |
| CAMSA | camsa_time_best | Seconds | Girls | 8 | 84 | 20.00 | 20.00 | 21.06 | 22.00 | 23.15 | 23.43 | 24.00 | 24.30 | 24.65 | 25.56 | 26.58 | 27.34 | 28.00 | 28.52 | 30.09 | 32.10 | 34.08 | 35.65 | 36.34 |
| CAMSA | camsa_time_best | Seconds | Girls | 9 | 84 | 18.00 | 19.15 | 20.00 | 20.50 | 21.00 | 22.00 | 22.55 | 23.00 | 23.47 | 24.00 | 25.52 | 25.99 | 27.00 | 27.81 | 28.00 | 29.48 | 30.15 | 30.62 | 32.32 |
| CAMSA | camsa_time_best | Seconds | Girls | 10 | 84 | 18.00 | 19.38 | 20.62 | 21.53 | 22.02 | 22.97 | 23.90 | 24.12 | 24.35 | 24.87 | 25.13 | 25.52 | 26.26 | 27.50 | 28.79 | 29.26 | 30.00 | 31.67 | 33.03 |
| CAMSA | camsa_time_best | Seconds | Girls | 11 | 85 | 17.25 | 18.04 | 19.56 | 20.00 | 20.00 | 20.05 | 20.44 | 21.00 | 21.01 | 22.00 | 22.11 | 22.94 | 23.15 | 23.64 | 24.50 | 25.56 | 27.16 | 28.79 | 29.84 |
| CAMSA | camsa_time_best | Seconds | Girls | 12 | 88 | 13.56 | 16.70 | 18.00 | 19.08 | 20.00 | 20.00 | 20.00 | 20.58 | 21.00 | 21.25 | 21.50 | 22.00 | 22.83 | 23.39 | 24.27 | 24.96 | 26.98 | 28.58 | 30.96 |
| CAMSA | camsa_time_best | Seconds | Boys | 8 | 84 | 15.74 | 17.00 | 17.89 | 18.00 | 18.08 | 19.00 | 19.18 | 20.00 | 20.31 | 21.80 | 22.70 | 23.09 | 24.27 | 24.43 | 27.03 | 27.90 | 28.10 | 29.48 | 30.37 |
| CAMSA | camsa_time_best | Seconds | Boys | 9 | 86 | 12.82 | 15.35 | 15.97 | 17.28 | 18.00 | 19.00 | 19.23 | 20.00 | 20.00 | 21.23 | 22.00 | 23.00 | 24.00 | 25.15 | 25.76 | 26.27 | 28.10 | 29.59 | 34.30 |
| CAMSA | camsa_time_best | Seconds | Boys | 10 | 87 | 11.96 | 12.92 | 14.50 | 16.03 | 17.50 | 18.29 | 19.00 | 20.10 | 21.00 | 21.00 | 21.81 | 23.14 | 24.79 | 25.16 | 26.09 | 27.00 | 28.94 | 30.40 | 32.00 |
| CAMSA | camsa_time_best | Seconds | Boys | 11 | 85 | 11.62 | 13.60 | 15.42 | 17.85 | 18.00 | 18.86 | 20.00 | 20.00 | 20.48 | 21.00 | 21.60 | 22.02 | 22.81 | 23.34 | 24.00 | 24.51 | 25.00 | 26.18 | 28.14 |
| CAMSA | camsa_time_best | Seconds | Boys | 12 | 76 | 10.65 | 12.35 | 13.25 | 16.00 | 17.04 | 18.94 | 19.61 | 20.00 | 20.00 | 20.36 | 21.12 | 22.00 | 22.72 | 24.09 | 25.00 | 26.00 | 27.96 | 28.61 | 31.47 |
| PACER | pacer_laps | Number of laps | Girls | 8 | 84 | 7.00 | 7.00 | 7.45 | 8.00 | 9.00 | 10.00 | 11.00 | 12.00 | 13.35 | 15.00 | 16.00 | 17.80 | 19.00 | 19.00 | 20.00 | 21.40 | 25.55 | 34.40 | 43.70 |
| PACER | pacer_laps | Number of laps | Girls | 9 | 84 | 7.00 | 8.00 | 8.45 | 10.00 | 11.00 | 12.90 | 14.00 | 14.00 | 16.00 | 17.00 | 18.65 | 20.80 | 22.00 | 25.10 | 28.50 | 31.40 | 34.00 | 42.50 | 46.85 |
| PACER | pacer_laps | Number of laps | Girls | 10 | 84 | 8.00 | 9.00 | 10.00 | 10.00 | 11.00 | 12.00 | 12.00 | 14.20 | 15.35 | 16.00 | 17.00 | 19.00 | 22.00 | 22.10 | 26.00 | 27.40 | 31.00 | 32.70 | 40.00 |
| PACER | pacer_laps | Number of laps | Girls | 11 | 85 | 10.00 | 11.40 | 13.00 | 15.00 | 15.00 | 16.20 | 17.00 | 18.00 | 19.00 | 20.00 | 21.00 | 22.00 | 23.00 | 24.80 | 29.00 | 30.20 | 32.00 | 33.00 | 41.60 |
| PACER | pacer_laps | Number of laps | Girls | 12 | 88 | 8.35 | 10.00 | 11.00 | 12.00 | 13.00 | 14.10 | 15.00 | 15.00 | 17.00 | 18.00 | 19.00 | 21.00 | 22.00 | 25.00 | 27.00 | 31.20 | 37.00 | 42.60 | 67.85 |
| PACER | pacer_laps | Number of laps | Boys | 8 | 84 | 7.00 | 8.00 | 9.00 | 9.00 | 11.00 | 12.90 | 15.00 | 16.00 | 18.35 | 19.00 | 19.00 | 20.00 | 20.00 | 22.00 | 24.00 | 27.00 | 29.00 | 34.40 | 47.80 |
| PACER | pacer_laps | Number of laps | Boys | 9 | 86 | 8.00 | 9.00 | 11.75 | 12.00 | 13.00 | 14.00 | 15.00 | 16.00 | 17.00 | 17.00 | 18.00 | 20.00 | 20.25 | 21.00 | 29.25 | 36.00 | 41.50 | 48.50 | 56.50 |
| PACER | pacer_laps | Number of laps | Boys | 10 | 87 | 7.30 | 9.60 | 12.00 | 14.00 | 15.00 | 16.80 | 18.00 | 19.00 | 20.00 | 21.00 | 22.00 | 23.00 | 25.00 | 27.20 | 31.00 | 33.60 | 36.30 | 41.00 | 48.70 |
| PACER | pacer_laps | Number of laps | Boys | 11 | 85 | 6.40 | 10.00 | 13.00 | 15.80 | 18.00 | 20.20 | 22.00 | 23.00 | 23.00 | 24.00 | 25.20 | 27.00 | 28.60 | 31.00 | 35.00 | 41.20 | 47.80 | 60.20 | 74.60 |
| PACER | pacer_laps | Number of laps | Boys | 12 | 76 | 10.75 | 15.00 | 18.00 | 20.00 | 22.50 | 23.00 | 25.00 | 26.00 | 27.75 | 30.00 | 31.25 | 32.00 | 39.00 | 42.50 | 50.00 | 53.00 | 55.00 | 62.00 | 64.00 |
| Average daily steps | step_average | Steps/day | Girls | 8 | 84 | 3,641 | 4,464 | 5,088 | 5,229 | 5,433 | 5,926.80 | 5,999.30 | 6,235.80 | 6,584.00 | 6,633 | 6,789 | 7,472.80 | 8,624.00 | 9,113.90 | 9,971.50 | 10,365 | 11,432 | 11,991 | 14,479 |
| Average daily steps | step_average | Steps/day | Girls | 9 | 84 | 5,194 | 5,347 | 5,613 | 6,021 | 6,227 | 6,891 | 7,208 | 7,694 | 8,204 | 8,374 | 8,691 | 9,368 | 9,636 | 10,158 | 10,640 | 11,120 | 11,480 | 12,348 | 12,545 |
| Average daily steps | step_average | Steps/day | Girls | 10 | 83 | 4,415 | 4,933 | 5,240 | 6,070 | 6,337 | 6,491 | 6,671 | 6,865 | 7,183 | 7,480 | 7,851 | 8,279 | 8,799 | 9,383 | 10,027 | 11,289 | 11,904 | 12,579 | 12,952 |
| Average daily steps | step_average | Steps/day | Girls | 11 | 85 | 4,136 | 5,108 | 5,889 | 6,369 | 6,501 | 6,743 | 6,954 | 7,042 | 7,493 | 7,946 | 8,330 | 9,562 | 10,362 | 10,699 | 11,298 | 11,943 | 12,226 | 12,799 | 14,255 |
| Average daily steps | step_average | Steps/day | Girls | 12 | 88 | 4,566 | 5,511 | 5,798 | 6,103 | 6,381 | 6,582 | 7,180 | 7,345 | 8,016 | 8,520 | 9,542 | 9,938 | 10,356 | 11,101 | 11,800 | 12,156 | 13,594 | 14,096 | 15,069 |
| Average daily steps | step_average | Steps/day | Boys | 8 | 83 | 4,965 | 5,162 | 6,442 | 6,859 | 7,139 | 8,048 | 8,605 | 8,795 | 8,999 | 9,618 | 9,786 | 10,214 | 10,821 | 11,549 | 11,966 | 12,468 | 13,455 | 14,199 | 15,699 |
| Average daily steps | step_average | Steps/day | Boys | 9 | 84 | 4,396 | 4,875 | 6,012 | 6,863 | 7,544 | 8,500 | 8,846 | 9,346 | 9,795 | 10,178 | 10,397 | 10,601 | 11,112 | 12,127 | 12,388 | 12,953 | 13,142 | 13,913 | 15,168 |
| Average daily steps | step_average | Steps/day | Boys | 10 | 86 | 4,396 | 5,439 | 5,913 | 6,505 | 7,200 | 8,008 | 9,194 | 9,674 | 10,190 | 10,880 | 11,113 | 11,553 | 12,524 | 13,072 | 13,383 | 14,068 | 14,760 | 15,659 | 16,854 |
| Average daily steps | step_average | Steps/day | Boys | 11 | 84 | 4,803 | 5,659 | 6,687 | 7,365 | 8,004 | 8,414 | 9,066 | 9,379 | 9,738 | 9,998 | 10,588. | 11,020 | 11,221 | 12,452 | 12,884 | 13,627 | 14,007 | 14,548 | 16,414 |
| Average daily steps | step_average | Steps/day | Boys | 12 | 74 | 4,230 | 5,371 | 6,730 | 7,446 | 7,892 | 8,643 | 8,946 | 9,633 | 9,858 | 10,319 | 10,945. | 11,199 | 11,479 | 12,159 | 12,991 | 13,494 | 14,457 | 15,306 | 16,316 |
| Plank | plank_time | Seconds | Girls | 8 | 84 | 15.77 | 21.22 | 23.97 | 25.00 | 29.56 | 35.80 | 37.05 | 39.40 | 41.70 | 44.50 | 46.00 | 46.99 | 52.87 | 60.00 | 67.72 | 75.47 | 88.29 | 105.43 | 221.00 |
| Plank | plank_time | Seconds | Girls | 9 | 84 | 23.85 | 28.00 | 29.00 | 32.05 | 33.97 | 35.98 | 37.00 | 38.19 | 41.93 | 44.50 | 47.00 | 48.20 | 51.96 | 54.10 | 58.00 | 61.91 | 72.10 | 86.10 | 105.95 |
| Plank | plank_time | Seconds | Girls | 10 | 84 | 23.24 | 30.00 | 33.45 | 35.60 | 38.97 | 41.98 | 43.72 | 48.00 | 50.00 | 52.00 | 52.95 | 54.83 | 56.99 | 62.95 | 68.97 | 72.80 | 80.24 | 85.47 | 91.70 |
| Plank | plank_time | Seconds | Girls | 11 | 85 | 19.20 | 28.00 | 33.60 | 39.00 | 41.16 | 42.67 | 45.55 | 47.00 | 48.99 | 53.00 | 56.00 | 57.76 | 60.00 | 67.14 | 73.90 | 78.53 | 89.17 | 101.52 | 147.00 |
| Plank | plank_time | Seconds | Girls | 12 | 88 | 20.35 | 24.91 | 29.03 | 33.40 | 36.75 | 38.12 | 42.00 | 43.00 | 47.15 | 49.59 | 55.00 | 60.09 | 63.21 | 65.03 | 69.93 | 72.60 | 80.39 | 91.07 | 106.94 |
| Plank | plank_time | Seconds | Boys | 8 | 84 | 23.15 | 28.30 | 32.45 | 36.60 | 41.31 | 48.02 | 50.52 | 58.09 | 59.79 | 60.00 | 69.67 | 71.95 | 73.91 | 82.48 | 88.52 | 101.98 | 125.22 | 133.00 | 174.28 |
| Plank | plank_time | Seconds | Boys | 9 | 86 | 23.24 | 27.48 | 36.25 | 39.84 | 42.00 | 47.00 | 48.75 | 54.17 | 58.25 | 59.00 | 60.00 | 62.00 | 64.55 | 69.00 | 76.17 | 83.01 | 90.22 | 98.41 | 140.75 |
| Plank | plank_time | Seconds | Boys | 10 | 87 | 27.69 | 35.38 | 38.00 | 45.20 | 47.00 | 48.86 | 52.28 | 56.00 | 58.88 | 60.00 | 60.00 | 65.88 | 74.93 | 78.03 | 84.94 | 92.89 | 111.60 | 137.38 | 177.90 |
| Plank | plank_time | Seconds | Boys | 11 | 85 | 18.23 | 26.00 | 31.98 | 35.00 | 39.00 | 43.13 | 48.40 | 54.60 | 57.80 | 60.00 | 70.38 | 76.30 | 78.21 | 87.66 | 92.22 | 102.19 | 109.40 | 124.69 | 153.20 |
| Plank | plank_time | Seconds | Boys | 12 | 76 | 16.51 | 20.50 | 32.75 | 41.11 | 47.00 | 50.00 | 55.31 | 57.00 | 59.87 | 63.05 | 69.58 | 74.00 | 78.38 | 82.48 | 85.88 | 93.00 | 102.00 | 111.53 | 130.06 |

Note. Percentiles were calculated empirically from the observed data for each assessment component, sex, and age combination. For CAMSA, the shortest completion time across the two attempts was used; therefore, lower values indicate better performance. For PACER, average daily steps, and plank duration, higher values indicate better performance. CAMSA and plank values are reported in seconds, PACER values as number of laps, and average daily steps as steps/day. Empirical percentile estimates are reported to two decimal places; step counts use commas as thousands separators and periods as decimal separators.
