# Supplementary material for: Age- and sex-specific reference values for physical literacy in Colombian children: a GAMLSS analysis of the CAPL-2
Source: Front Sports Act Living. 2026 Sep 7;8:1963012. doi: 10.3389/fspor.2026.1963012 (PMC13590728; doi:10.3389/fspor.2026.1963012)
Supplement: Supplementary file 4 [file Table4.docx]

**Supplementary Material S4. STROBE checklist for reporting a cross-sectional study**

*Age- and sex-specific reference values for physical literacy in Colombian children: a GAMLSS analysis of the CAPL-2*

Checklist mapped to the final submitted manuscript. STROBE recommendations are paraphrased for concise reporting. Page locations correspond to the current manuscript version and may require updating after journal typesetting.

| **Section** | **Item** | **STROBE reporting recommendation** | **Status** | **Verification / comment** |
| --- | --- | --- | --- | --- |
| Title and abstract | 1a | Indicate the study design with a commonly used term in the title or abstract. | Yes | The abstract explicitly identifies the study as cross-sectional and multicenter. |
| Title and abstract | 1b | Provide an informative and balanced summary of what was done and what was found. | Yes | The abstract summarizes the rationale, objective, sample, GAMLSS analysis, main findings, and interpretation. |
| Introduction | 2 | Explain the scientific background and rationale for the investigation. | Yes | The manuscript describes the physical literacy construct, CAPL-2, cross-cultural use, the need for contextual reference distributions, and the Colombian evidence gap. |
| Introduction | 3 | State the specific objectives, including any prespecified hypotheses when applicable. | Yes | The primary objective concerns age- and sex-specific CAPL-2 reference values; secondary objectives address correspondence with official categories and empirical percentiles for selected raw components. |
| Methods | 4 | Present key elements of the study design early in the Methods section. | Yes | The study is described as observational, cross-sectional, and multicenter. |
| Methods | 5 | Describe the setting, locations, and relevant dates, including recruitment and data-collection periods. | Partial | The geographical setting, participating schools, and school-day assessment context are reported. The final manuscript does not state the calendar period or dates of participant recruitment/data collection. |
| Methods | 6 | Describe eligibility criteria, participant sources, and methods of participant selection. | Yes | Eligibility and exclusion criteria, purposive selection of cities/schools, random selection of eligible children within schools, and the six geographical subregions are reported. |
| Methods | 7 | Clearly define outcomes, exposures, predictors, potential confounders, effect modifiers, and diagnostic criteria when relevant. | Yes | CAPL-2 domains, total score, age, sex, raw assessment components, scoring structure, and modelled outcomes are defined. Confounder adjustment was not part of the study objective. |
| Methods | 8 | Describe data sources and measurement methods for each variable of interest and comparability across groups when relevant. | Yes | Questionnaires, pedometer, CAMSA, PACER, plank, CAPL-2 scoring, evaluator training, and standardized administration procedures are described. |
| Methods | 9 | Describe efforts made to address potential sources of bias. | Yes | Random selection of children within participating schools, evaluator training/calibration, standardized field procedures, data verification, and explicit acknowledgement of purposive site selection are reported. |
| Methods | 10 | Explain how the study size was determined. | Yes | The analytical sample of 843 children is reported, together with the statement that no separate a priori sample-size calculation was performed specifically for construction of percentile reference values. |
| Methods | 11 | Explain how quantitative variables were handled in the analyses and, if applicable, why groupings were chosen. | Yes | Age was entered as a continuous explanatory variable in GAMLSS; models were fitted separately by sex. Empirical raw-component percentiles were summarized by age and sex. |
| Methods | 12a | Describe all statistical methods, including methods for confounding control when applicable. | Yes | Data cleaning, CAPL-2 scoring, GAMLSS fitting, candidate distributions, GAIC/SBC model selection, percentile estimation, category correspondence, and empirical percentile calculation are described. |
| Methods | 12b | Describe methods used to examine subgroups and interactions. | Yes | Models were fitted separately for girls and boys and age-specific percentiles were estimated. No interaction model was required because sex-specific modelling was prespecified. |
| Methods | 12c | Explain how missing data were addressed. | Yes | Missing data were not imputed; each analysis used the valid observations available for the corresponding domain, total score, or assessment component. |
| Methods | 12d | For cross-sectional studies, describe analytical methods that account for the sampling strategy when applicable. | Not applicable | The study did not use a probability-weighted national sampling design; survey weights were therefore not applied. The manuscript explicitly avoids nationally representative claims. |
| Methods | 12e | Describe sensitivity analyses, when performed. | Not applicable | No separate sensitivity analysis was prespecified. Candidate models were assessed using convergence, GAIC, SBC, and plausibility of percentile curves. |
| Results | 13a | Report numbers of individuals at each relevant stage of the study. | Yes | The reference sample includes 843 children, and valid observations are reported for each CAPL-2 domain and the total score. |
| Results | 13b | Give reasons for non-participation or missing observations at each stage, when available. | Partial | Outcome-specific completeness is quantified, but reasons for incomplete component data are not reported in the final manuscript. |
| Results | 13c | Consider use of a participant flow diagram. | Not applicable | A flow diagram is not essential for this single-time-point cross-sectional analysis because participant counts and valid-case numbers are clearly reported. |
| Results | 14a | Describe characteristics of study participants and relevant exposure/confounder information. | Yes | Age range, sex distribution, geographical distribution, and total sample size are reported. |
| Results | 14b | Indicate the number of participants with missing data for each variable of interest. | Yes | Valid n and percentages are reported for each CAPL-2 domain and total score, allowing outcome-specific missingness to be determined. |
| Results | 15 | For cross-sectional studies, report outcome events or summary measures. | Yes | Selected GAMLSS models, age- and sex-specific percentiles, category-threshold percentile positions, and empirical percentiles for raw components are reported. |
| Results | 16a | Provide main estimates and their precision when relevant, making clear which estimates are adjusted or unadjusted. | Yes | The principal outputs are GAMLSS-derived and empirical percentile estimates. Conventional confidence intervals are not the primary estimand for this reference-distribution analysis. |
| Results | 16b | Report category boundaries when continuous variables are categorized. | Yes | The original CAPL-2 interpretive categories are retained unchanged, and the percentile position of the minimum score required to enter each category is reported. |
| Results | 16c | When relevant, translate relative-risk estimates into absolute risk for a meaningful time period. | Not applicable | The study does not estimate relative risks, incidence rates, or time-to-event outcomes. |
| Results | 17 | Report other analyses performed, such as subgroup or sensitivity analyses. | Yes | Sex-specific models, candidate-model comparisons, category-correspondence analyses, and empirical raw-component percentiles are reported. |
| Discussion | 18 | Summarize key results with reference to the study objectives. | Yes | The Discussion opens by summarizing the age- and sex-specific reference values, GAMLSS approach, and complementary relationship with the original CAPL-2 categories. |
| Discussion | 19 | Discuss limitations, including potential sources of bias or imprecision. | Yes | The manuscript addresses purposive selection of sites, limits on national representativeness, the cross-sectional design, and reduced stability of extreme empirical percentiles in smaller age-sex subgroups. |
| Discussion | 20 | Provide a cautious overall interpretation considering objectives, limitations, multiplicity of analyses, and other relevant evidence. | Yes | Interpretation is contextual, avoids diagnostic and nationally representative claims, compares findings with prior studies, and acknowledges conceptual and sampling limitations. |
| Discussion | 21 | Discuss the generalizability (external validity) of the findings. | Yes | The percentiles are explicitly framed as reference values from a geographically diverse multicenter Colombian sample rather than nationally representative norms, and replication in independent samples is recommended. |
| Other information | 22 | State the source of funding and the role of funders, if any. | Yes | The manuscript states that the authors did not receive financial support for the research, authorship, or publication of the article. |

Abbreviations: CAPL-2, Canadian Assessment of Physical Literacy, second edition; GAMLSS, generalized additive models for location, scale, and shape; GAIC, generalized Akaike information criterion; SBC, Schwarz Bayesian criterion. Status: Yes = adequately reported; Partial = reporting is present but one detail remains incomplete; Not applicable = item does not apply to the present analysis.
