# Supplementary material for: Age- and sex-specific reference values for physical literacy in Colombian children: a GAMLSS analysis of the CAPL-2
Source: Front Sports Act Living. 2026 Sep 7;8:1963012. doi: 10.3389/fspor.2026.1963012 (PMC13590728; doi:10.3389/fspor.2026.1963012)
Supplement: Supplementary file 5 [file Table5.docx]

**Supplementary Table S5. Age- and sex-specific analytical sample sizes for each CAPL-2 outcome**

| **Domain** | **Variable** | **Sex** | **Age 8** | **Age 9** | **Age 10** | **Age 11** | **Age 12** | **Total n** |
| --- | --- | --- | --- | --- | --- | --- | --- | --- |
| Physical Competence | pc_score | Girls | 84 | 82 | 81 | 83 | 87 | 417 |
| Physical Competence | pc_score | Boys | 84 | 83 | 85 | 84 | 76 | 412 |
| Daily Behavior | db_score | Girls | 82 | 83 | 82 | 83 | 84 | 414 |
| Daily Behavior | db_score | Boys | 83 | 83 | 84 | 84 | 71 | 405 |
| Motivation and Confidence | mc_score | Girls | 84 | 82 | 84 | 82 | 87 | 419 |
| Motivation and Confidence | mc_score | Boys | 84 | 86 | 87 | 83 | 75 | 415 |
| Knowledge and Understanding | ku_score | Girls | 84 | 84 | 83 | 84 | 88 | 423 |
| Knowledge and Understanding | ku_score | Boys | 84 | 85 | 87 | 84 | 76 | 416 |
| Total CAPL-2 score | capl_score | Girls | 82 | 83 | 82 | 83 | 83 | 413 |
| Total CAPL-2 score | capl_score | Boys | 83 | 83 | 84 | 83 | 70 | 403 |

Note. Values represent the number of participants with valid data available for each CAPL-2 outcome within each age- and sex-specific stratum. Because the calculation of each CAPL-2 domain and the total score required different assessment components, analytical sample sizes varied slightly across outcomes. Missing data were not imputed. CAPL-2: Canadian Assessment of Physical Literacy, second edition.
